# Blackwater Fever in Pregnancy With Severe Falciparum Malaria: A Case of Imported Malaria From Nigeria to the United Kingdom During the COVID-19 Pandemic

**DOI:** 10.7759/cureus.20170

**Published:** 2021-12-04

**Authors:** Cledervern Brebnor Des Isles, Anisha Chitrakar, Heena Patel, Mark Finney

**Affiliations:** 1 Obstetrics and Gynaecology, University Hospitals of Leicester NHS Trust, Leicester, GBR

**Keywords:** imported malaria, sickle cell trait, re-emerging disease, pyrexia, pregnancy, severe falciparum, congenital anomaly, cardiac, covid-19, blackwater fever

## Abstract

We present the case of imported malaria in pregnancy to the United Kingdom (UK) from Nigeria, where a 28-year-old primigravida presented to our maternity assessment unit (MAU) with complaints of pyrexia, rigors and passing dark coloured urine. She gave a travel history of recent migration from Nigeria 10 days before presenting to our emergency department.

She initially became unwell five days after her arrival with general malaise and myalgia. On day six, she developed lower abdominal pain and observed that her urine was dark in colour. This prompted her to contact her general practitioner (GP). Treatment for a urinary tract infection was initiated by the GP after a phone consultation in keeping with COVID-19 contingency guidance, and the patient was prescribed antibiotics for three days.

She presented to the emergency department two days after completing the course of antibiotics where she complained of worsening pelvic pain, reduced foetal movements and passing black urine. She was treated as suspected COVID-19 and red flag sepsis. Obstetric review led to investigation and diagnosis of severe malaria in pregnancy, which was accompanied by blackwater fever (BWF). The patient recovered after three doses of artesunate. An ultrasound scan of the foetus revealed a congenital cardiac anomaly, which had not been detected in an earlier scan. There was no evidence of congenital malaria in the neonate after delivery.

There are several novel aspects in this case as maternal mortality in severe *Plasmodium falciparum* can be significantly high. Those who survive the disease in pregnancy are also known to develop several complications such as intrauterine death and preterm labour. There was also the component of blackwater fever, which is a rare event associated with severe malaria, and it also has a mortality rate. Significant in her medical history was a diagnosis of the sickle cell trait, and we postulate that this feature gave an added protection from the complications of severe malaria in pregnancy as well as blackwater fever.

## Introduction

The onset of the COVID-19 pandemic has led to other infectious diseases with similar presentations, such as malaria, being underdiagnosed. Common to both are fever, headache, chills, arthralgia, vomiting and diarrhoea. Prior to the pandemic, any patient presenting with a fever from a malaria-endemic country or those with a travel history to such regions would be under high suspicion of this potentially fatal parasitic infection. With the emergence of the COVID-19, it has become the main differential for patients who present with pyrexia. This has led to the potential to overlook other causes of a patient’s presentation, such as malaria, with the potential of poorer outcomes due to a delay in diagnosis [1].

In 2019, 1,719 cases of malaria were reported in the United Kingdom (UK) [2], all of which were imported from endemic areas. There was no specific data about pregnant women amongst the data mentioned as most of the literature was based on isolated case reports. Nevertheless, the disease is known to have high mortality and morbidity for both mother and foetus [3].

The WHO malaria report in 2020 voiced that significant improvement has been made in the fight against the parasite over the past 20 years [3]. However, there is now a concern amongst public health officials that reduced funding for malaria programmes, due to a significant proportion of resources being focused on COVID-19, may lead to a resurgence of this disease amongst at-risk populations [4].

According to the WHO, 10,000 maternal deaths occur each year from malaria. Pregnant women are 10 times more likely to contract malaria [5], and they are three times more likely to suffer from severe disease as a result of malaria infection when compared with their non-pregnant counterparts [6]. Malaria is caused by five different species of the plasmodia parasite and is transmitted by the female *Anopheles* mosquito [6]. Amongst them, *Plasmodium falciparum* is the most virulent and is responsible for 20% of mortality in pregnancy [6]. Apart from this, the risks of stillbirth, spontaneous miscarriage and intrauterine growth restriction are associated with malaria infection in pregnancy. Hence, pregnant women should be advised about traveling to such areas and if possible defer travel until after delivery or ensure that safe effective prophylactic medication is used [7]. It is also recommended by the WHO that preventative measures should still be undertaken even though antimalarial prophylactic medication is being used since malaria can still be contracted. These include the use of bed nets and mosquito repellents and remaining indoors after sunset. It is also recommended to wear loose-fitting clothing, long trousers and long sleeves and remain indoors from dusk to dawn [8].

## Case presentation

A 28-year-old primigravida presented at 32 weeks with a five-day history of pelvic pain, lethargy and fever. She indicated that she arrived in the United Kingdom as a student 10 days earlier and developed myalgia, abdominal pain and fever that started five days after her arrival. On day six, she developed worsening lower abdominal pains and observed that her urine was a dark colour. At this time, she had a virtual phone consultation with her GP, where she complained of suprapubic pain and the passage of dark urine at each void. The GP diagnosed her with a urinary tract infection and prescribed a three-day course of antibiotics. Despite the antibiotics, she reported that her urine changed to a black colour and that the fever and lower abdominal pain persisted. She also had a one-day history of reduced foetal movements. The patient had no significant medical history, except for uterine fibroids. There was also no significant family or drug allergy history. She denied contact with any ill person.

She also denied any previous infection with malaria, HIV or hepatitis. She was classified as a late booker in the United Kingdom (UK) and grouped as high risk since there was no documented evidence of previous antenatal care. The dating of the pregnancy was based on her menstrual cycle, which was regular as there was no dating scan.

Clinical examination and investigations

Upon review, the patient was febrile (38°C) and tachycardic (HR: 132 bpm) with a blood pressure reading of 132/80 mmHg. The cardiovascular and respiratory examinations were unremarkable. There was localised tenderness in the suprapubic region upon abdominal palpation; however, there was no organomegaly.

An antenatal foetal cardiotocography (CTG) was performed using the Dawes Redman analysis. The criteria for foetal well-being were not met due to a foetal tachyarrhythmia of 190 bpm.

She was admitted, and a full septic screen was performed in line with treatment for red flag sepsis. A COVID nasopharyngeal swab was also taken and analysed with real-time polymerase chain reaction (RT-PCR), along with full booking blood (HIV, *Treponema*, and hepatitis B and C), haemoglobinopathy screen (thalassaemia and sickle cell test) and glucose-6-phosphate dehydrogenase (G6PD) deficiency test. There was a high suspicion of malaria based on the history of dark-coloured urine along with the patient’s travel history; hence, a rapid malaria antigen test was requested along with thick and thin films for confirmation of any parasitaemia. Initial laboratory investigations were consistent with active haemolysis (Table 1). A repeat full blood test two hours later showed a pattern in keeping with haemolysis as there was a continued trend of anaemia, thrombocytopenia and raised bilirubin. In particular, there was a drop in haemoglobin to 99 g/L, haematocrit 0.294 L/L, platelet count 64 × 10^9^/L, and red cell count 3.55 × 10^12^/L, and bilirubin level increased to 89 umol/L.

**Table 1:** Laboratory test results

Laboratory test	Value	Reference range
Full blood count		
White cell count	6.3 × 10^9^/L	4–11 × 10^9^/L
Red cell count	3.87 × 10^12^/L	3.9–5.60 × 10^12^/L
Haemoglobin	110 g/L	115–165 g/L
Haematocrit	0.316 L/L	0.370–0.470 L/L
Platelet count	82 × 10^9^/L	140–400 × 10^9^/L
Urea and electrolytes		
Sodium	135 umol/L	133–146 umol/L
Potassium	4 umol/L	3.5–5.3 umol/L
Urea	3.9 umol/L	2.5–7.8 umol/L
Creatinine	50 umol/L	60–120 umol/L
Liver function test		
Total protein	5 g/L	60–80 g/L
Albumin	30 g/L	35–50 g/L
Alkaline phosphatase	95 IU/L	30–113 IU/L
Alanine transaminase	12 IU/L	2–53 IU/L
Total bilirubin	59 umol/L	0–21 umol/L
Coagulation profile		
INR	1	
PT	14.1 seconds	
aPTT	29.5 seconds	
aPTT ratio	1.0	

The COVID-19 PCR test was negative, along with G6PD, HIV, and hepatitis B and C; however, the sickle cell test was positive, and haemoglobin electrophoresis confirmed that the patient was a sickle cell carrier.

On the routine haematology blood film, platelet numbers appeared low. Ring-form trophozoites were seen with morphology consistent with *Plasmodium falciparum* (multiple infected red cells and double clots), and no other life stages were seen. Parasitaemia was 3%-4%. These findings were consistent with the rapid diagnostic test and confirmed the diagnosis of severe malaria in pregnancy.

Urine microscopy was also carried out to rule out haematuria after the collection of a midstream urine sample using aseptic techniques. Manual microscopy of the urine sample reported a white blood cell count of 5 × 10^6^/L and no epithelial cell, hence excluding haematuria. Haptoglobin testing was not performed. Throughout admission, her renal function was preserved despite having macroscopic haemoglobinuria as her estimated glomerular filtration rate (eGFR) remained at >/90. The same trend was noted with the electrolyte balance of the patient, which remained normal throughout her sequelae. There was no evidence of any coagulopathy on admission (Table 1), and this trend continued at subsequent follow-up investigations.

Diagnosis

The timeline on the patient’s arrival to the United Kingdom was not initially forthcoming from the patient despite her being quite unwell clinically when she arrived at the hospital. However, the differential of malaria was chosen as a high priority due to the patient’s history. The initial diagnosis of malaria was made on the background clinical presentation and findings, along with an initial rapid antigen malaria test. This included ethnicity, a recent arrival in the United Kingdom and poor/absence of antenatal care. The diagnosis was later further established by confirmation of *P. falciparum* parasitaemia on blood film. The clinical approach was to widely exclude causes of pyrexia rather than focus only on the now common COVID-19 diagnosis. Malaria took first priority as the patient’s urine on close inspection was observed to be the classic Coca-Cola colour as described in the literature for blackwater fever (BWF), along with her complaint of pyrexia in the MAU triage. She also had mild conjunctival icterus, which progressively worsened as her total bilirubin increased due to haemolysis of red blood cells.

After confirmation of the diagnosis with laboratory evidence, the patient was again approached and asked about her travel history and sick contacts. She then confided that her husband was also sick at home with a fever, and they arrived from Nigeria 10 days before.

Treatment

The patient was admitted to high-dependency unit (HDU) care, was started on intravenous artesunate 12 hours after her initial admission and made a good recovery after three doses. Twenty-four hours after the administration of the first dose of artesunate, there were no parasites in malarial blood films. She was then switched to oral therapy, where she completed a full course of artesunate.

Her general care was tailored to the treatment of severe malaria in pregnancy based on guidance from the Royal College of Obstetrics and Gynaecology green top guideline [9]. There was careful fluid management to reduce the risk of pulmonary oedema and daily blood films for parasitaemia and four hourly blood glucose monitoring for hypoglycaemia. The foetal tachycardia resolved as there was a steady improvement in the maternal status.

Even though our main differential and diagnosis was malaria in pregnancy, the patient was concomitantly treated with meropenem prophylactically to prevent gram-negative septicaemia, which is commonly seen in severe malaria infection. She received a total of 24 hours of meropenem and continued on a five-day course of oral cefuroxime after review by our infectious disease specialist. Blood cultures yielded no bacterial growth after two days and subsequent days, up to five days, post culture.

The patient was discharged after a five-day admission to the hospital and with a plan to follow up in one month to ensure favourable response.

Follow-up and outcome

Maternal Outcome

In the follow-up blood film one month later, no malaria parasites were seen.

Foetal Outcome

An anomaly scan was also performed as an inpatient at 32 + 4, which concluded no obvious foetal defects. A growth scan at 37 + 2 weeks showed appropriate growth; however, there was an abnormal four-chamber heart view with a disproportion between the right and the left ventricle. A foetal echocardiogram confirmed a right ventricle/left ventricle disproportion with hypoplastic aortic arch. Prenatal microarray analysis confirmed a male foetus with no significant copy number changes detected. This was consistent with previous results from a rapid FISH, which was done previously. The growth remained appropriate, and she was offered induction of labour at 38 + 5 due to the foetal cardiac anomaly, which resulted in an emergency caesarean section. At delivery, the neonate was started on prostaglandin E1 (PGE1) and transferred to the congenital heart centre for augmentation surgery. A screen for congenital malaria on day two of life confirmed that there was no in utero transfer of any malarial parasites. Examination of the placenta showed no histological abnormality.

## Discussion

In this case, the initial diagnosis at the emergency department was COVID-19 with a differential diagnosis of red flag sepsis. It was after review by the obstetric team that consideration for a diagnosis of malaria was made, and the patient was treated and investigated for this. Both diseases can have a similar presentation, such as fever, headache, shortness of breath and fatigue. The complications of severe malaria are also similar to COVID-19: these include acute respiratory distress syndrome (ARDS), septic shock and multiorgan failure [10]. Although new and poorly understood, the evidence thus far suggests that patients from malaria-endemic areas have a protective effect against COVID-19, thus accounting for the low infection rates of the disease in African countries [11,12]. It is predicted that the reduced expression of the ACE2 receptor in populations with polymorphism of the D-allele of ACEI/D may play a protective role against COVID-19 [13]. This is a double-edged sword, as whilst there may be protection against the COVID-19 virus, there has been a struggle to prevent the resurgence of the parasitic infection in these countries due to the derailment of funding from anti-malaria programmes to the COVID-19 pandemic. In our case, where malaria is an imported condition, the patient’s diagnosis and treatment were also impacted directly by pandemic and our national COVID-19 contingency plans, when she was first diagnosed and treated for a urinary tract infection by her GP after a phone consultation and subsequently treated primarily for COVID-19 in the emergency department. Bearing this in mind, we need to retain a high level of suspicion for malaria in any patient who presents from an endemic country or has a travel history to such regions, with fever. We also need to consider that there can be co-infection with COVID-19 in febrile patients. There is also the need to also consider that patients with severe malaria are at increased risk of developing concomitant bacterial sepsis, which also increases the mortality rate [14]. Hence, the treatment of severe malaria needs to include suitable antimicrobial agents as was done in our patient.

Although significant inroads have been made against malaria in the past 20 years, very few studies have been done on malaria in pregnancy due to ethical restrictions; hence, the disease remains poorly understood. It is known, however, that the most common maternal complication is anaemia along with placental sequestration of the parasite. This can trigger a pathological process that contributes to adverse outcomes such as low birth weight, intrauterine growth restriction and preterm delivery. A standard definition for severe malaria has been a longstanding problem; however, in pregnancy, a parasitaemia of greater than 2% places patients at risk of developing a severe infection, and such women should be treated with the severe malaria protocol, with HDU or intensive care unit admission [9,15]. As such, respiratory distress syndrome, pulmonary oedema, hypoglycaemia (<2.2 mmol/L) and gram-negative septicaemia are common findings in women with complicated or severe malaria [16].

In our case, the patient had a clinical presentation of blackwater fever (BWF), but no other features of complicated malaria. Apart from the black-coloured urine, which is due to haemoglobinuria, her diagnosis of severe malaria was confirmed by laboratory findings such as hyperparasitaemia (3%-4%), thrombocytopenia (platelet count: 64 × 10^9^/L) and hyperbilirubinemia (89 umol/L). To account for this, we explore the patient factors that may have contributed to this outcome.

Blackwater fever (BWF) is a rare event in severe malaria and is a representation of acute intravascular haemolysis [17]. It is mainly associated with infection by *Plasmodium falciparum* but has also been documented in *Plasmodium vivax*, *Plasmodium malariae* and mixed infections [18]. The exact mechanism and pathophysiology of BWF are poorly understood; however, it has significant mortality [19]. Apart from malaria, the condition is known to be associated with glucose-6-phosphate dehydrogenase (G6PD) deficiency, treatment with quinine or concomitant infection with other viruses or bacteria [20,21]. Although it is not pathognomonic, acute renal failure has been reported in some cases of severe malaria with blackwater fever, and in such instances, it is associated with high mortality [22].

Her kidney function and electrolytes remained in the normal range, which suggested preservation of her renal function despite the passage of dark-coloured urine. We postulate here that since our patient was a carrier of the sickle cell trait, this may have offered protection against developing complications from the severe malaria infection. The literature indicated that patients with inherited haemoglobinopathies such as sickle cell trait (haemoglobin AS), thalassaemias, haemoglobin C, and G6PD deficiency have a protective component against death from falciparum malaria [23]. The mechanism of how this protection is proffered is not well understood; however, amongst the inherited haemoglobinopathies, the sickle cell trait offers the most protection against malaria [24]. Also, despite the patient not giving a history of previous malarial infection, it is also likely that she had acquired partial immunity [23,24], which is common in those who live in endemic areas.

At the follow-up growth scan, the discovery of a foetal cardiac defect was concerning as the previous scan reported no congenital anomaly. Consideration was given as to the cause of this diagnosis, whether it was congenital or induced from the severe malaria infection or was it from the treatment with artesunate. Careful evaluation of the drug for treatment was undertaken before deciding to treat it with artesunate. It is the recommended drug for severe malaria in pregnancy and is safe in all trimesters. From previous studies, artesunate is not associated with an increased risk of miscarriage, stillbirth or congenital anomalies [25,26]. There are very few studies that focused on the cardiac effect in severe malaria; however, they have all indicated that the parasite does affect heart function [27]. As we reflected, we concluded that it was a congenital abnormality that was not picked up before as there were no other features of complications in the neonate post-infection, such as intrauterine growth restriction. This was confirmed at birth with a normal screen for congenital malaria and no sequestration of parasites in the placenta at histology.

## Conclusions

Malaria continues to be a parasitic disease that can have devastating effects and continues to be a challenge globally. Pregnant women are more susceptible to malaria due to the immunocompromised state of pregnancy and are at greater risk of developing severe foetal and maternal complications.

The omission of malaria as a possible differential in any patients who present with pyrexia from an endemic region or in those with travel history to such areas can have a consequential impact on the outcome of the patient’s well-being, including death. On the contra side, malaria is highly treatable, and when it is recognised and diagnosed in a timely manner, the outcome is favourable. Prompt diagnosis and appropriate treatment are then crucial to prevent morbidity and fatal outcomes.

Although this was not a feature in our patient, it is also crucial to remember that malaria can co-exist with sepsis, and they must be distinguished from each other as not doing so can again have fatal outcomes secondary to untreated septicaemia.

In conclusion, we need to be mindful of the impact the COVID-19 pandemic has on global healthcare including the lack of funding on other infectious diseases and hence a threat of a resurgence of such cases. It is therefore imperative that clinicians should remain open-minded when dealing with pyrexia in any patient and not be overshadowed by the now common COVID-19 diagnosis.
